# Devilliers and Chailley on Auscultation in Pregnancy

**Published:** 1842-11

**Authors:** 


*Devilliers and Chailly on Auscultation in Pregnancy.*—To determine the position of the maximum intensity of the double sounds of the fœtal heart, we divide the abdomen into four parts by two lines crossing one another perpendicularly at the umbilicus, one falling from the extremity of the xiphoid cartilage to the symphysis pubis, the other running horizontally from side to side. In cranial presentations, the sounds will be heard most intensely below, in presentation of the pelvic extremity above the transverse line; the precordial region being nearer to the head than the pelvis. It is very difficult to diagnose presentations of the trunk, inasmuch as the sounds being heard below the transverse line, they may be confounded with cranial positions. The maximum intensity of the sounds on the right or left of the perpendicular line indicates a right or left position of the head. The more distinctly the sounds are heard in the median line, the greater is the probability that the abdomen of the child is directed anteriorly. In the opposite position the sounds are heard more clearly in a lateral situation. But these signs, especially in the latter case, are very difficult, and we are often obliged to have recourse to other means to assist in the diagnosis. A table is added, according to which a correct diagnosis was formed by auscultation in 223, a false one in 68 cases of pregnancy. The large proportion of the latter was owing to the difficulty in determining the anterior or posterior, and right or left positions of the child.—*London Med. Gaz.*, from *Revue Médicale.*

